# Injurious mechanical ventilation in the normal lung causes a progressive pathologic change in dynamic alveolar mechanics

**DOI:** 10.1186/cc5940

**Published:** 2007-06-12

**Authors:** Lucio A Pavone, Scott Albert, David Carney, Louis A Gatto, Jeffrey M Halter, Gary F Nieman

**Affiliations:** 1 Department of Surgery, SUNY Upstate Medical University, 750 East Adams St Syracuse, NY 13210, USA; 2 Memorial Health University Medical Center, 4700 Waters Ave Savannah, GA 31404, USA; 3 Department of Biological Sciences, SUNY Cortland, P.O. Box 2000 Cortland, NY 13045, USA

## Abstract

**Introduction:**

Acute respiratory distress syndrome causes a heterogeneous lung injury, and without protective mechanical ventilation a secondary ventilator-induced lung injury can occur. To ventilate noncompliant lung regions, high inflation pressures are required to 'pop open' the injured alveoli. The temporal impact, however, of these elevated pressures on normal alveolar mechanics (that is, the dynamic change in alveolar size and shape during ventilation) is unknown. In the present study we found that ventilating the normal lung with high peak pressure (45 cmH_2_0) and low positive end-expiratory pressure (PEEP of 3 cmH_2_O) did not initially result in altered alveolar mechanics, but alveolar instability developed over time.

**Methods:**

Anesthetized rats underwent tracheostomy, were placed on pressure control ventilation, and underwent sternotomy. Rats were then assigned to one of three ventilation strategies: control group (*n *= 3, *P*_control _= 14 cmH_2_O, PEEP = 3 cmH_2_O), high pressure/low PEEP group (*n *= 6, *P*_control _= 45 cmH_2_O, PEEP = 3 cmH_2_O), and high pressure/high PEEP group (*n *= 5, *P*_control _= 45 cmH_2_O, PEEP = 10 cmH_2_O). *In vivo *microscopic footage of subpleural alveolar stability (that is, recruitment/derecruitment) was taken at baseline and than every 15 minutes for 90 minutes following ventilator adjustments. Alveolar recruitment/derecruitment was determined by measuring the area of individual alveoli at peak inspiration (*I*) and end expiration (*E*) by computer image analysis. Alveolar recruitment/derecruitment was quantified by the percentage change in alveolar area during tidal ventilation (%*I *– *EΔ*).

**Results:**

Alveoli were stable in the control group for the entire experiment (low %*I *– *EΔ*). Alveoli in the high pressure/low PEEP group were initially stable (low %*I *– *EΔ*), but with time alveolar recruitment/derecruitment developed. The development of alveolar instability in the high pressure/low PEEP group was associated with histologic lung injury.

**Conclusion:**

A large change in lung volume with each breath will, in time, lead to unstable alveoli and pulmonary damage. Reducing the change in lung volume by increasing the PEEP, even with high inflation pressure, prevents alveolar instability and reduces injury. We speculate that ventilation with large changes in lung volume over time results in surfactant deactivation, which leads to alveolar instability.

## Introduction

The treatment of acute lung injury and the acute respiratory distress syndrome remains largely supportive, in the form of mechanical ventilation. However, mechanical ventilation has been implicated in the development of ventilator-induced lung injury (VILI) and is felt to significantly contribute to the high-mortality-associated acute respiratory distress syndrome [1]. A growing interest in VILI has developed with evidence that mortality can be reduced when lung-protective ventilatory strategies are employed [2,3]. VILI is of particular concern in patients with acute respiratory distress syndrome because of the heterogeneous pattern of injury, with areas of acutely injured lung adjacent to areas of normal lung morphology. It is believed that the injured regions are rendered stiff and noncompliant due to the accumulation of pulmonary edema and deactivation of surfactant. The pressures required to inflate the injured lung areas are consequently much higher than those needed to inflate the more compliant regions of healthy lung. This difference results in shunting of excessive tidal volume into the healthy lung, causing lung injury by either alveolar overdistension [4-6] or recruitment/derecruitment [7-11].

To simulate the ventilator-induced injury that occurs in normal regions of the lung, we employed a commonly used model of injurious mechanical ventilation (IMV) (high tidal volume and low positive end-expiratory pressure (PEEP)). Although alveolar recruitment/derecruitment is most commonly associated with acute lung injury [8-11], it is possible that the large lung volume excursion created by the high inflation pressures and the low PEEP might cause repetitive alveolar recruitment/derecruitment in the normal lung. Indeed, the temporal effects of IMV on alveolar mechanics are unknown.

In the present study, we utilized *in vivo *microscopy to directly measure subpleural alveolar mechanics (that is, dynamic changes in alveolar size and shape during tidal ventilation) in the living animal ventilated with high tidal volume and both low PEEP and high PEEP.

## Materials and methods

### Surgical preparation

Adult, male Sprague–Dawley rats weighing between 298 g and 548 g were anesthetized with intraperitoneal ketamine (90 mg/kg) and xylazine (10 mg/kg) at the onset of the procedure and as needed to maintain surgical anesthesia. A tracheostomy was established with a 2.5 mm pediatric endotracheal tube. Paralysis was then achieved with intravenous pancuronium (0.8 mg/kg) and the rats were placed on pressure control ventilation with 50% oxygen delivered via a Galileo ventilator (Hamilton Medical, Reno, NV, USA). Baseline ventilator settings included a control pressure (*P*_control_, the pressure applied above that of the PEEP during the inspiratory phase) of 14 cmH_2_O and a PEEP of 3 cmH_2_O.

A carotid arterial catheter was placed for blood gas analysis (model ABL5; Radiometer Inc., Copenhagen, Denmark) and inline measurement of systemic arterial pressure (TruWave™; Baxter Healthcare Corp., Irvine, CA, USA). The internal jugular vein was cannulated for fluid and drug infusion. Fluid resuscitation was performed with a 1 cm^3 ^bolus of lactated Ringer's solution when the mean arterial pressure fell below 60 mmHg. Rats were then placed on zero PEEP and a midline sternotomy was performed with removal of the right third to sixth ribs. The lung volume history was standardized by generating a single inflation from zero PEEP to a peak pressure of 25 cmH_2_O at a constant rate of inflation (3 cmH_2_O/s; Galileo Ventilator™ and PV Tool™; Hamilton Medical, Inc.).

### Experimental groups

Following surgical instrumentation, the rats were placed on the ventilator and assigned to one of three ventilatory strategies: control group (*n *= 3), maintained on the baseline ventilatory strategy (*P*_control _= 14 cmH_2_O, PEEP = 3 cmH_2_O); high pressure/low PEEP (HP/LP) group (*n *= 6), *P*_control _increased to 45 cmH_2_O and PEEP maintained at 3 cmH_2_O; and high pressure/high PEEP (HP/HP) group (*n *= 5), *P*_control _increased to 45 cmH_2_O and PEEP increased to 10 cmH_2_O.

Concomitant with the initiation of the experimental ventilatory strategies, the respiratory rate was set to 20 breaths/min for all groups. Time 0 was designated as the time immediately following initiation of the experimental ventilatory strategy. Hemodynamic, lung function, and *in vivo *microscopic data were recorded at baseline and every 15 minutes after initiation of the experimental protocol. The protocol was terminated after 90 minutes.

### *In vivo *microscopy

A microscopic coverslip mounted on a ring was lowered onto the pleural surface and the lung was held in place by gentle suction (≤5 cmH_2_O) at end inspiration for placement of an *in vivo *videomicroscope (epi-objective microscope with epi-illumination, Olympus Model BXFM; (Olympus America Inc. Melville, NY USA). At each timepoint, the apparatus was reattached to the lung, and thus a different cohort of alveoli was sampled at each timepoint. The lung tissue in the coverslip apparatus was filmed field-by field from one edge of the coverslip to the other. Microscopic images of alveoli were viewed at a final magnification of 130× with a color video camera (model CCD SSC-S20; SONY, Tokyo, Japan) and recorded on Pinnacle Studio Plus software (Pagasus Imaging Corporation Tampa, FL). Each field measured 1.22 × 10^6 ^μm^2 ^and was filmed throughout five complete tidal breaths.

### Image analysis of alveoli

**Figure 1:**
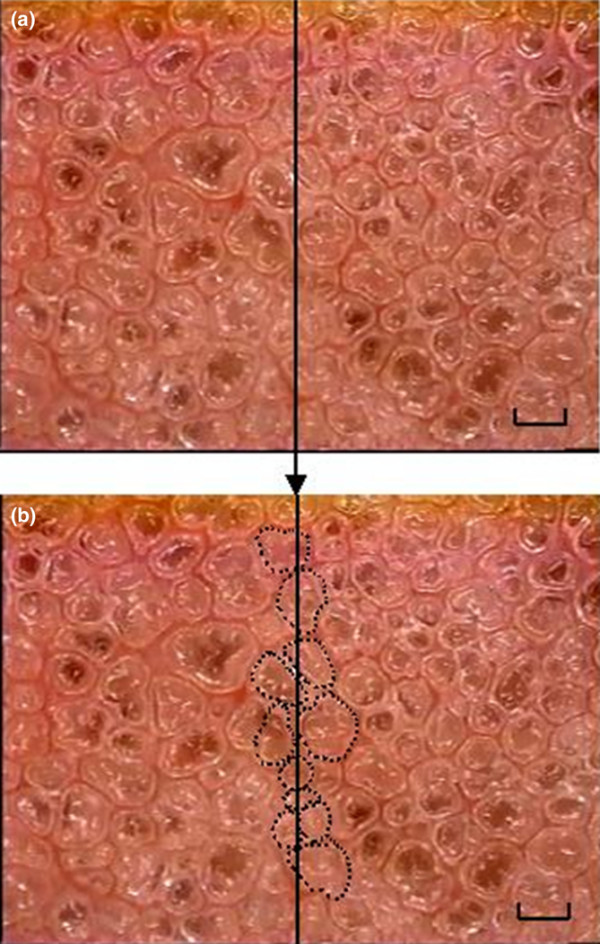
Randomization of alveoli for measurement of alveolar stability. The percentage change in alveolar area between peak inspiration and end expiration.** (a) **For each microscopic field analyzed, a vertical line bisecting the field was drawn. **(b) **Each alveolus that contacted this bisecting line was chosen for analysis of alveolar stability. Bar = 100 μm.

We analyzed the alveolar mechanics by replaying the video frame-by-frame and capturing still images of individual alveoli at peak inspiration (*I*) and end expiration (*E*). For each visual field, the subset of alveoli analyzed consisted of those that contacted a vertical line bisecting the visual field, representing approximately 10 alveoli per field (Figure 1). Five microscopic fields were analyzed in each animal at each timepoint. A mean of 38 alveoli (range 8–65) per timepoint per animal was analyzed. The large range was due to alveolar collapse (atelectasis) in the HP/LP group.

**Figure 2:**
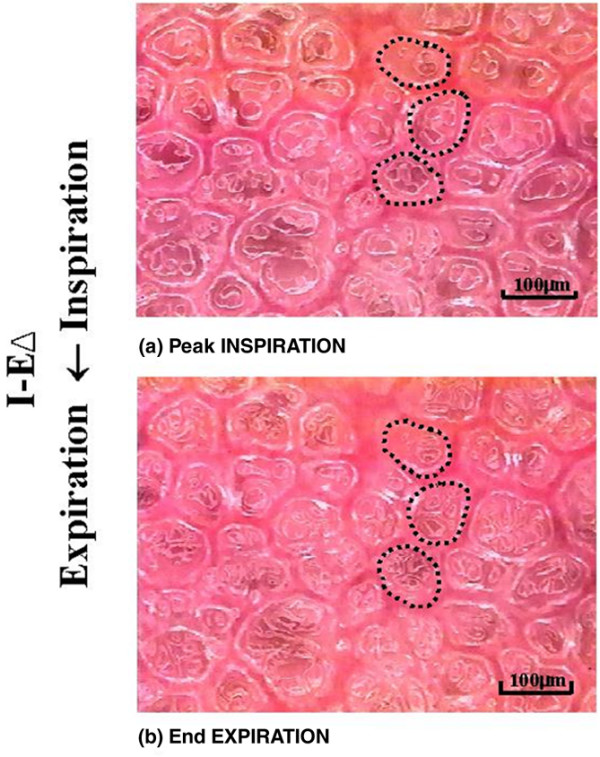
Image analysis measurement of alveolar stability. *In vivo *photomicrographs of the same microscopic field at **(a) **peak inspiration and **(b) **end expiration. Individual alveoli were outlined and the area at peak inspiration (*I*) and end expiration (*E*) was measured using image analysis software. Alveolar stability was assessed by the percentage change in the area of individual alveoli from *I *to *E *(%*I *– *EΔ*).

Measurements of alveolar area were made by manually tracing the outer wall of individual alveoli at both *I *and *E *(Figure 2). Computer image analysis (Empire Imaging Systems; Image Pro, Syracuse, NY, USA) was then used to measure the cross-sectional area of each traced alveolus. The degree of alveolar stability – the change in alveolar size during tidal ventilation (from *I *to *E*) – was quantified by calculating the percentage change in alveolar area from *I *to *E *(%*I *– *EΔ*).

### Hemodynamic and lung function measurements

Arterial blood gases, systemic arterial pressures, and pulmonary parameters (exhaled tidal volume and peak airway pressure) were recorded at baseline and then at 15-minute intervals for two hours. Pulmonary parameters were calculated inline by the Galileo ventilator (Hamilton Medical): the peak airway pressure was, by definition, the highest airway pressure measured during the breath cycle.

### Necropsy

At necropsy, the right lung (which was filmed during the protocol) was excised and its bronchus cannulated. The lung was inflated with 10% formalin by gravity to a pressure of 25 cmH_2_O. After 24 hours, the tissue was blocked in paraffin and serial sections were made for staining with hematoxylin and eosin. The slides were reviewed at high magnification (400×). Additionally, a tissue sample from the left lung was sharply dissected free of nonparenchymal tissue. The sample was weighed before and every 24 hours after incubation at 65°C. This was repeated until there was no weight change over a 24-hour period, at which time the samples were deemed to be dry. Lung water was expressed as a wet to dry weight ratio.

### Statistics

All values are reported as the mean ± standard error of mean. Significant differences between groups were determined by analysis of variance and significant differences within groups by a repeated-measures analysis of variance. Whenever the *F *ratio indicated significance, a Newman–Keul's test was used to identify the individual differences. Significance was assumed when the probability of the null hypothesis being true was less than 5% (*P *< 0.05).

### Vertebrate animals

Experiments described in this study were performed in accordance with the National Institutes of Health guidelines for the use of experimental animals in research. The protocol was approved by the Committee for the Humane Use of Animals at our institution.

## Results

### Hemodynamic and pulmonary function

Hemodynamic and pulmonary parameters are presented in Table 1. The HP/LP group was the only group to develop significant hypotension at 90 minutes compared with baseline, with no difference in fluid administration between the groups (9.3 ± 0.7 ml in the control group, 12.6 ± 2.9 ml in the HP/LP group, 9.0 ± 1.9 ml in the HP/HP group, *P *= not significant). The peak airway pressure was significantly higher in the HP/HP and HP/LP groups as compared with the control group (Table 1). The tidal volume was significantly greater in the HP/LP group as compared with either the control group or the HP/HP group (Table 1).

**Table 1:** Hemodynamic and pulmonary parameters

	Baseline	15 minutes	30 minutes	45 minutes	60 minutes	75 minutes	90 minutes
**Control group**							
Mean arterial pressure (mmHg)	102 ± 17	111 ± 10	123 ± 7	138 ± 0	143 ± 8	124 ± 10	105 ± 20
pH	7.32 ± 0.05	7.32 ± 0.10	7.30 ± 0.10	7.26 ± 0.01	7.26 ± 0.02	7.26 ± 0.05	7.26 ± 0.02
PCO_2 _(mmHg)	28 ± 9	40 ± 1	36 ± 0	37 ± 4	34 ± 5	29 ± 2	23 ± 5
PO_2 _(mmHg)	241 ± 81	316 ± 18	325 ± 36	298 ± 16	331 ± 37	334 ± 10	340 ± 12
Tidal volume (ml/kg)	15.0 ± 5.9	7.8 ± 1.3	7.8 ± 1.3	7.8 ± 1.3	6.9 ± 1.6	7.9 ± 1.4	9.9 ± 1.8
Peak pressure (cmH_2_O)	16 ± 0	15 ± 1	16 ± 0	16 ± 0	16 ± 0	16 ± 0	16 ± 0
Intravenous fluid (ml)		9.3 ± 0.7					
**High pressure/low PEEP group**							
Mean arterial pressure (mmHg)	92 ± 13	79 ± 10	89 ± 10	86 ± 9*	71 ± 11*	64 ± 11*	55 ± 10^#^
pH	7.25 ± 0.06	7.39 ± 0.05^#^	7.37 ± 0.04^#^	7.33 ± 0.03	7.31 ± 0.04	7.24 ± 0.04	7.20 ± 0.03
PCO_2 _(mmHg)	30 ± 6	23 ± 5*^†^	22 ± 4	21 ± 4	17 ± 3*	16 ± 3	15 ± 4
PO_2 _(mmHg)	187 ± 32	241 ± 37	260 ± 42	240 ± 54	244 ± 59	251 ± 54	232 ± 45
Tidal volume (ml/kg)	12.2 ± 5.9	31.4 ± 5.5*^#†^	30.3 ± 3.9*^#†^	27.7 ± 2.6*^#†^	38.4 ± 10.8*^#†^	40.0 ± 13.2^#†^	39.0 ± 9.2*^#†^
Peak pressure (cmH_2_O)	17 ± 0*	39 ± 4*	45 ± 1*	45 ± 1*	45 ± 1*	45 ± 1*	46 ± 2*
Intravenous fluid (ml)		12.6 ± 2.9					
**High pressure/high PEEP group**							
Mean arterial pressure (mmHg)	105 ± 23	94 ± 13	100 ± 16	85 ± 14*	84 ± 20*	83 ± 16	66 ± 14
pH	7.40 ± 0.05	7.27 ± 0.04	7.32 ± 0.08	7.28 ± 0.03	7.27 ± 0.04	7.23 ± 0.06^#^	7.22 ± 0.09^#^
PCO_2 _(mmHg)	32 ± 3	44 ± 3	33 ± 7	26 ± 4	25 ± 3	19 ± 1	15 ± 1^#^
PO_2 _(mmHg)	263 ± 32	262 ± 40	315 ± 14	307 ± 27	317 ± 20	315 ± 11	317 ± 17
Tidal volume (ml/kg)	12.9 ± 1.4	8.6 ± 2.4^#^	9.6 ± 2.5^#^	9.6 ± 2.6^#^	9.6 ± 2.6^#^	9.2 ± 2.4^#^	9.2 ± 2.4^#^
Peak pressure (cmH_2_O)	17 ± 0*	50 ± 3*	50 ± 6*	50 ± 3*	50 ± 3*	50 ± 3*	50 ± 6*
Intravenous fluid (ml)		9.0 ± 1.9					

Data presented as the mean ± standard error. PCO_2 _(partial pressure of carbon dioxide); PO_2 _(partial pressure of oxygen). *P < 0.05 versus control group, ^#^*P *< 0.05 versus baseline, ^†^*P *< 0.05 versus the high pressure/high positive end-expiratory pressure (PEEP) group.

### Alveolar mechanics

**Figure 3:**
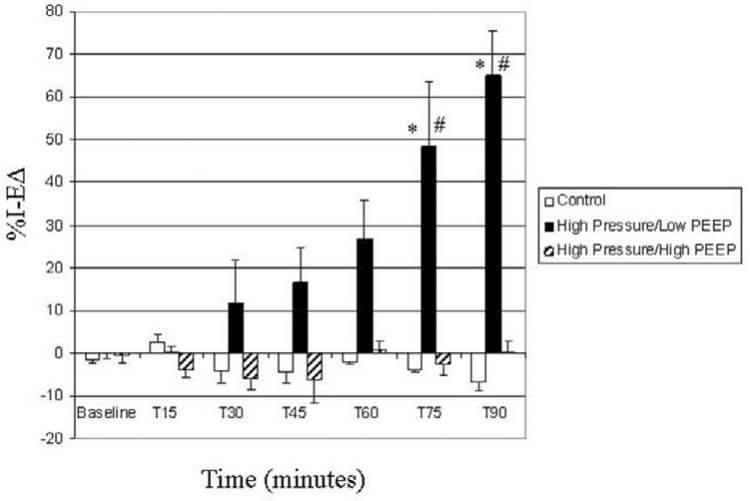
Alveolar stability. Expressed as the percentage change in alveolar area between peak inspiration and end expiration (%*I *– *EΔ*). Data are the mean ± standard error. *P < 0.05 versus control group and high pressure and high positive end-expiratory pressure (PEEP) group, #*P *< 0.05 versus baseline.

Alveoli were stable (minimal change in size during tidal ventilation, low %*I *– *EΔ*) in all groups on baseline ventilator settings (*P*_control _= 14 cmH_2_O, PEEP = 3 cmH_2_O) (Figure 3). Alveoli were also stable in the control group for the entire 90-minute protocol (see Additional file 1). Alveoli in the HP/LP group were stable initially but became unstable with time (Figure 3) (see Additional file 2). The application of additional PEEP (HP/HP group) prevented alveolar instability through the entire protocol (Figure 3).

### Arterial blood gases

**Figure 4:**
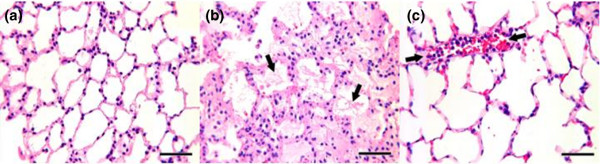
Rat lung stained with hematoxylin and eosin. **(a) **Control group. **(b) **High pressure and low positive end-expiratory pressure group (arrows indicate fibrinous deposits in the alveolar lumen). **(c) **High pressure and high positive end-expiratory pressure group (arrows indicate inflammatory cells in the vascular compartment). Bar = 50 μm

Arterial blood gas values are also presented in Table 1. Despite the marked alveolar instability (Figure 3) and lung injury (Figure 4) in the HP/LP group, the arterial PO_2 _(partial pressure of oxygen) was actually higher at 90 minutes than at baseline (Table 1). The PCO_2 _(partial pressure of carbon dioxide) in all groups trended downward throughout the experiment.

### Gross morphology, histology, and lung water determination

Lungs in the HP/LP group at necropsy appeared cherry red, with areas of hemorrhagic consolidation evident at the pleural surface. Lungs in the control and HP/HP groups appeared pink, without evidence of hemorrhage on the pleural surface.

The histologic assessment was analyzed qualitatively, and the pictures displayed (Figure 4) were selected by our histologist (LAG) as representative of each group. The control group revealed normal morphology with few inflammatory cells and minimal evidence of pulmonary edema (Figure 4a). The HP/LP group exhibited thickened alveolar walls, an abundance of inflammatory cells, and alveolar edema indicated by the presence of fibrinous material within the alveolar lumen (Figure 4b). These histopathologic changes were mitigated with the addition of PEEP in the HP/HP group. Only a moderate amount of edema and relatively few inflammatory cells were seen in the pulmonary parenchyma of this group (Figure 4c).

There was a numerically higher, but not statistically different, increase in lung edema, determined by the lung wet/dry weight ratio, in the HP/LP group (wet to dry weight ratio: control group = 5.39 ± 0.72, HP/LP group = 6.35 ± 0.71, HP/HP group = 5.62 ± 0.21, *P *= not significant).

## Discussion

To our knowledge this is the first study to directly observe and quantify subpleural alveolar mechanics in healthy lungs exposed to IMV over time. We found that alveoli were initially stable but became unstable with time. Increasing PEEP prevented the development of gross pathologic changes and of alveolar recruitment/derecruitment in spite of high peak airway pressure and volume. We postulate that the large volume change with IMV resulted in surfactant deactivation [12-17], which caused alveolar instability [8,18-21]. Large changes in lung volume could deactivate surfactant by any of the following mechanisms: direct inhibition [16,22], increased surfactant release from type II cells with subsequent removal from the alveolar surface [12-15,23], and/or increased vascular permeability resulting in edema-induced surfactant deactivation [16,24-26]. The PEEP may have prevented alveolar instability either by reducing surfactant deactivation or by maintaining end-expiratory pressure above the alveolar collapse point [14,16]. The fact that injury was greater in lungs with unstable alveoli suggests that alveolar instability (atelectrauma) exacerbates the damage caused by high lung volume (volutrama) [27].

Previous work has demonstrated that normal alveoli do not change size appreciably during positive pressure ventilation with either physiologic [8,19,21] or high tidal volumes [20] and airway pressures. We have demonstrated in surfactant deactivation animal models of acute respiratory distress syndrome that alveoli are unstable even with physiologic tidal volumes and that alveolar recruitment/derecruitment causes lung damage [8,18,19,21]. Unlike previous studies in which the lung was injured by Tween instillation (deactivates surfactant) [8,18,19,21], the present experiment was performed on normal lungs. In this study we demonstrated that, even with a very large tidal volume and low PEEP, normal alveoli, at least in the two dimensions we can see with our *in vivo *microscopic technique, do not change size appreciably during ventilation. In the previous experiments we only maintained high lung volume for a very short period of time [20,28]. In contrast, in the present experiment IMV was maintained for 90 minutes and, eventually, the lungs developed abnormal alveolar mechanics with unstable alveoli.

How could a large change in lung volume occur without a change in either the size or number of alveoli? What is the critical factor or factors that convert an alveolus with normal stable alveolar mechanics into an unstable alveolus with abnormal mechanics? Why was no deterioration in gas exchange associated with unstable alveoli and lung injury?

The mechanism by which the normal lung changes volume at the alveolar level is poorly understood. The following discussion highlights some of the possible mechanisms of lung volume change that would explain our consistent finding that normal alveoli do not change size appreciably during tidal ventilation.

There are substantial data to support the hypothesis that lung volume change is not simply due to a balloon-like isotropic change in alveolar volume. Macklin suggested that the alveolar size changes little during lung volume change and that the increase in lung volume is accommodated by changes in volume of the alveolar ducts [29]. Daly and colleagues corroborated these findings utilizing *in vivo *microscopy, and showed that the alveolar duct was the anatomical structure that changes size during tidal ventilation [30]. Carney and colleagues demonstrated in normal lungs inflated from functional residual capacity (FRC) to 80% total lung capacity (TLC) that the lung volume change was due to 'normal' alveolar recruitment (that is, normal lung volume change is due to alveoli opening and closing, not to alveoli getting larger and smaller) [28]; this hypothesis was corroborated by Escolar and colleagues using stereologic techniques [31]. Other workers have shown that the lung volume change is caused by folding and unfolding of the alveolus, similar to a paper bag [23].

If any or all of these mechanisms are responsible for the change in lung volume, this could explain our finding that there is little change in alveolar size even with a large change in lung volume. If the lung changes volume by changes in the size of the alveolar duct, this would not be visible due to the limited depth of field of our *in vivo *microscope. Likewise, bag-like folding and unfolding of the alveolus would be in the third dimension, invisible to our *in vivo *microscopic view. Regardless of the mechanism, our data clearly show a distinct difference in alveolar mechanics in the normal versus the acutely injured lung, which leads us to our next question.

What are the critical factors that convert an alveolus with normal stable alveolar mechanics into an unstable alveolus with abnormal mechanics? We postulate that the large volume change deactivates pulmonary surfactant [12-17,22,24,26], which would cause alveoli to become unstable [8,18-21]. In a similar study, Verbrugge and colleagues demonstrated that rats ventilated with high tidal volume and low PEEP had altered surfactant composition, with a significant decrease in the ratio of functional to nonfunctional surfactant [16]. This change in surfactant composition resulted in reduced surfactant function as measured by a pulsating bubble surfactometer. In addition to the altered composition of surfactant, Verbrugge and colleagues also measured a significant increase in bronchoalveoalar lavage fluid protein concentration, which can deactivate surfactant directly [16,25,32]. Moreover, those authors demonstrated that the addition of 10 cmH_2_O of PEEP reduced or prevented all of the above changes [16]. Other workers have suggested that large tidal volumes increase the rate of surfactant turnover, effectively 'wearing out' the surface film at a very high rate [12-15].

The data demonstrating that high tidal volume and low PEEP ventilation inhibits pulmonary surfactant [12-17,22,24-26,32], combined with our data showing that subpleural alveoli become unstable following surfactant deactivation [8,18-21], support the hypothesis that surfactant deactivation is the mechanism of alveolar instability in this study. This brings us to our final question: why was no deterioration in gas exchange associated with unstable alveoli and lung injury?

In the present study, oxygenation was not significantly different at 90 minutes between any of the groups, even though only the HP/LP group(high pressure/low PEEP) had unstable alveoli. How can a lung with alveoli that collapse at end expiration oxygenate as well as a lung with patent alveoli throughout the ventilatory cycle? We hypothesize that surfactant-deficient, unstable alveoli are forced open during lung inflation due to the exceeding large tidal volume and inflation pressure with IMV. While inflated, these alveoli would exchange gas and load oxygen into the arterial blood.

Baumgardner and colleagues utilized a fluorescence-quenching PO_2 _probe placed inside the distal aorta [33]. They demonstrated that PO_2 _fluctuated breath-by-breath; the magnitude of the PO_2 _oscillations was dependent upon the degree of alveolar instability (that is, the amount of collapse and re-expansion with each breath). Adjustments in ventilation (for example, respiratory rate, PEEP, etc.) that reduced alveolar instability also reduced PO_2 _fluctuation. In a subsequent study, Syring and colleagues demonstrated that increasing the respiratory rate was as effective as increasing the PEEP to improve arterial PO_2 _in a rabbit saline lavage model of acute respiratory distress syndrome [34]. Interestingly, a respiratory rate of 24/minute with a PEEP of 3.5 cmH_2_O was as effective at maintaining PO_2 _as a respiratory rate of 7/minute and a PEEP of 14 cmH_2_O. In our HP/LP group, the respiratory rate was 20/minute and the PEEP was 3 cmH_2_O, very similar to the ventilator settings in the Syring and colleagues study that yielded good oxygenation.

We hypothesize that high inflation pressure would further improve oxygenation in noncompliant alveoli by forcing more collapsed alveoli open. The plateau pressure in Syring and colleague's study was 28 cmH_2_O, as compared with the peak inspiratory pressure of 45 cmH_2_O in the present HP/LP group. We speculate that even though alveoli in our HP/LP group were very 'stiff', they were recruited during inspiration due to the high inflation pressure – and the rapid respiratory rate kept them inflated for a sufficient length of time to adequately oxygenate the blood. Although forcing surfactant-deficient unstable alveoli open with each breath will improve oxygenation in the short run, it will cause a tremendous amount of mechanical damage to the pulmonary parenchyma (VILI) and will significantly exacerbate morbidity and mortality [18,21].

## Critique of the model

### Respiratory rate, inspratory:expiratory ratio, and carbon dioxide

Numerous factors have been implicated in the development of VILI. For example, it has been shown that alterations in the respiratory rate [33,35] and the inspiratory:expiratory ratio [36] can impact VILI. For this reason, we employed a protocol that standardized the respiratory rate and inspiratory:expiratory ratio between the groups (respiratory rate, 20/min; inspiratory:expiratory ratio, 1:2). Additionally, it has been shown that hypercapnic acidosis protects against VILI [37,38] and that, conversely, hypocapnic alkalosis can injure isolated rabbit lungs [39]. There was no significant difference in the pH or partial pressure of carbon dioxide values between the groups at the end of the protocol (Table 1). The development of alveolar instability, as well as gross and histologic changes, is therefore reflective of the differences in ventilatory pressures and volumes, not of the acid–base status.

### Microscopic artifact

Although there are methodologic problems with our *in vivo *microscopic technique, it is the only tool available to directly observe the behavior of alveoli in a living animal. Utilizing electron microscopy, we have previously confirmed that the subpleural structures measured are true alveoli [19]. Concern exists, however, regarding whether subpleural alveolar mechanics might differ from those in other regions of the lung. Subpleural alveoli are, indeed, structurally different from alveoli in deeper regions of the lung, in that they are not completely surrounded by other alveoli. In other words, one wall of a subpleural alveolus is always adjacent to the visceral pleura rather than another alveolus. This anatomic arrangement may serve to lessen the structural support provided by alveolar interdependence, causing subpleural alveoli to become unstable sooner than those within the interior of the lung.

Our microscope's limited depth of field (70 μm) restricts our analysis of alveolar mechanics to only two dimensions. Regardless of this limitation, we clearly demonstrate that alveolar mechanics are dramatically altered in two dimensions following exposure to IMV.

To maintain the same microscopic field during tidal ventilation, gentle suction (≤5 cmH_2_O) must be applied to hold the lung tissue under the coverslip. This amount of suction is within the range of normal intrapleural and transpulmonary pressures. In a previous study we compared the alveolar size at *E *and *I *as well as the stability of normal alveoli during mechanical ventilation with and without suction [19]. We demonstrated that suction slightly but significantly increased the alveolar size at both *I *and *E *and stabilized the alveolus. These changes were very subtle, with %*I *– *EΔ *being 1.1% in the suction group and 8.3% in the nonsuction group [19]. This slight change in alveolar size with ventilation was in stark contrast to the marked change in alveolar size that occurred following prolonged exposure in the HP/LP group. These data therefore demonstrate that suction did not fix the alveolar volume, demonstrate that normal alveolar stability is not an artifact of suction, and demonstrate that suction does not prevent the development of alveolar instability.

### Image analysis of alveoli

The measurement was performed in a nonblinded fashion. Unfortunately, this may have introduced some bias. If minimal bias were introduced, however, we feel very confident that this would not change our results significantly since there was such a large difference in %*I *– *EΔ *between groups.

## Key messages

• A large change in lung volume with each breath will, in time, lead to unstable alveoli and pulmonary damage.

• Reducing the change in lung volume by increasing the PEEP, even with high inflation pressure, prevents alveolar instability and reduces lung injury.

• We speculate that ventilation with large changes in lung volume over time results in surfactant deactivation, which leads to alveolar instability.

## Abbreviations

*E *= end expiration; HP/HP = high pressure and high positive end-expiratory pressure; HP/LP = high pressure and low positive end-expiratory pressure; %*I *– *EΔ *= percentage change in alveolar area; *I *= peak inspiration; IMV = injurious mechanical ventilation; *P*_control _= control pressure; PEEP = positive end-expiratory pressure; PO_2 _= partial pressure of oxygen; VILI = ventilator-induced lung injury.

## Competing interests

The authors declare that they have no competing interests.

## Authors' contributions

LAP conducted the experiments, analyzed and graphed the data, and wrote the first draft of the paper. JMH assisted LAP in conducting the experiments and editing the manuscript. LAG contributed to the experimental design, data analysis and interpretation, and performed the histologic analysis. DC contributed to the experimental design of the study, data analysis, and interpretation. SA assisted with manuscript drafting, data analysis and extensive editing. GFN contributed to the design and development of the protocol, data analysis and interpretation, and writing of the manuscript.

## Supplementary Material

Additional file 1 A movie file illustrating stable subpleural alveoli in the normal rat lung during dynamic tidal ventilation. Each sphere-shaped object is an inflated individual alveolus and there is minimal atelectasis (that is, the entire field is covered by inflated alveoli). Notice there is minimal alveolar movement (that is, alveoli are stable) during tidal ventilation, at least in the two dimensions that can be seen with our *in vivo *microscope technique. Click here for file

Additional file 2 A movie file demonstrating unstable subpleural alveoli in the injured rat lung during dynamic tidal ventilation. The red area without alveoli (that is, individual circles) shows diffuse atelectasis prior to inspiration. The individual alveoli (spheres) 'pop' open with inspiration and then quickly collapse with expiration. Notice that alveoli are very unstable and there is complete collapse of most alveoli during deflation and than reinflation during lung inflation – classic alveolar recruitment/derecruitment. Click here for file
